# Supplementary material for: Acceptance and compliance with micronutrient powder (MNP) among children aged 6–23 months in northern Nigeria
Source: PLOS Glob Public Health. 2022 Oct 17;2(10):e0000961. doi: 10.1371/journal.pgph.0000961 (PMC10022258; doi:10.1371/journal.pgph.0000961)
Supplement: S1 Dataset — (ZIP) [file pgph.0000961.s008.zip › S8_Dataset/Direct Observations.zip!/Direct Observations/190615_K_BK_SU_1_DO.pdf]

1.1.1 Instrument F –Direct Observation Form

<<Instrument F Direct Observation Form>>

PI Name: PragyaMathema,Stephen Kodish
Study Title: Formative research to develop an infant and young child feeding (IYCF) intervention with micronutrient powder (MNP) for children aged 6 – 23 months
PI Version/Date: Version1.0/January 24, 2015

Date: 19/06/2015 Location: Bakan Yadiya

Data Collector: Samira Usman

Main goals of this direct observation are to answer the following questions:

1. In this household, what would be the best way(s) to give nutritional supplements to young children? Why?

2. In this household, describe the food sharing that you observe throughout the meal. Are there any predictable patterns?

3. In this household, who is favored and how is this favoritism expressed during meal preparation and eating?

Household Composition Table (Fill out as you do data collection)

INITIALS	SEX (M/F)	AGE (estimated)	Relationship to household	Description
MM	F	20yrs	Mother	Yellow wrapper, red shirt and head tie
BB	M	9months	Child/son	Blue shirt and pant
BB2	M	3yrs	Son	Purple shirt and milk pant
FW	F	25 yrs	First wife	Ankara wrapper, top and head tie
MI	F	55 yrs	Mother in-law	Red and green wrapper, top and head tie (Ankara)
FI	M	61 yrs	Father in-law	Milk shadda and brown cap
DF	F	9 yrs	First wife daughter	Green wrapper, top and head tie (Ankara)
BDF	F	12yrs	Big daughter of the first wife	Blue jeans skirt, pink shirt and blue hijab
FF	F	29 YRS	Father	Blue long gown and trouser (shadda)

SDF	F	5 yrs	Daughter	Purple skirt and top

TIME	ACTOR INITIALS	RECIPIENT INITIALS	WHAT HAPPENED	FOOD	Reactivity
10:05	BB		Baby is sleeping		
10:10	BB2		The young child is playing		
10:15	BB		Baby still sleeping		
10:20	FW		The first wife just enter to greet me		R
10:25	MM		Mother washing clothes		
10:30	MM	BB2	Mother backing the baby		
10:35	MM		Mother still washing the clothes with the baby on her back		
10:40	MM	BB	Baby wakes up crying, the mother brought him down and gave him water	water	
10:45	MM		Mother sweeping her room and compound		R
10:50	BB2		The 3 yrs old boy is eating ground nut he bought outside and drinking water from the kettle	groundnut	
10:55	BB		Baby playing around the house		
11:00	FF		Father just enter, pick kettle entered toilet and went out		

TIME	ACTOR INITIALS	RECIPIENT INITIALS	WHAT HAPPENED	FOOD	Reactivity
	MM	BB	Mother is sitting breastfeeding the baby		
11:05	MM		Mother went and fetch raw sorghum in a calabash		
11:10	MM	BB	Mother sit and breast feed the baby		
11:15	MM	BB	Mother breast feeding the baby		
11:25	All members of the family		Everybody is sitting under the tree and the baby is playing		
11:30	BB BB2		The young boy is playing with the baby his brother		
11:35	MM	BB	Mother playing with the baby		
11:40	MM	BB	Mother is breast feeding the baby		
11:45	BB		Baby playing		
11:55	BB		Baby still playing on the ground		
12:00	MM	BB	Baby crying and the mother took him and breast feed him		
12:05	MM	BB	Mother is backing the baby		
12:10	BB2		The young boy is eating tuwon dawa (sorghum) and dried baobab leaves soup	Tuwo sorghum	
12:15	FW		First wife is pounding beans in a motter to remove the chaff. She washes the beans with water keep the grain and give their animals the water	beans	
12:20	BB		Baby is playing		
	SBF		The small girl is drinking fura in a silver cup	Fura (millet)	
	BDF		Big daughter of the first wife is pounding millet after she finished, she wash it with water		

TIME	ACTOR INITIALS	RECIPIENT INITIALS	WHAT HAPPENED	FOOD	Reactivity
12:25	MM	BB	Mother washes the baby's body and sat down to breast feed him		
12:30	BDF		The big daughter of first wife put the millet grains, she pound it into flour and sieve it, added water re - pound it into pastry.		
12:35	FW	BB	Baby is still on the first wife lap playing		
	DF		The girl is washing clothes		
	FI<br>MI		They are sleeping under the tree		
	MM	BB	Baby starts crying and he's mother took him and breast feed him		
12:40	BB		Baby is playing		
12:45	BB		Playing		
12:50	BB		Playing with his brother		
12:55	FW	BB	The first wife is picking the remaining chaff of the beans she pounded and took some and give the baby	Raw beans	
1:00	MM	BB	Mother is breast feeding the baby		
1:05	FF		Father came to the house holding the hand of the young boy (BB2), pick his clothes from the rope, fetch water in the bucket and enter toilet, the first wife follows him		
1:10	BB		Baby playing around the house		
1:15	FF		Father husband came and sit down under the tree, the baby is playing with his brother		
1:20	MM	BB	Baby is crying and the mother breast feeding him		

TIME	ACTOR INITIALS	RECIPIENT INITIALS	WHAT HAPPENED	FOOD	Reactivity
1:25	MM	BB	Mother breast feeding the baby		
1:30	FI		Father in-law playing with the his grandson and daughter, the grand daughter is scratching his back		
1:35	BB		Baby is playing		
1:40	FM	BB	Baby is crying, his mother bring pap and MNP pour it inside and give the first wife to give him, she look at me and doesn't want to take the pap, he is crying he took some and leave the remaining. The pap is not thick it is watery	Pap and MNP	R
1:45	MM	BB	Mother breast feeding the baby		
1:50	BB		Baby is sleeping		
1:55	BB		Sleeping		
2:00	BB		Sleeping		
2:05	NW	MM	A woman (neighbor) came in and ask the mother to please give her some tuwo I they have, she gave her and she left	tuwo	
2:10	BB		Baby still sleeping, the mother handle him to the first wife and went to fetch water		
2:15	MM	BB	Mother is backing the baby		
2:20	BB		Baby still sleeping		
2:25	MM	BB	Mother takes the baby to his grandmother and lay him down on a local bed of the grand mother		
1:35	MM		Mother went and pray. She comes back with sorghum that she left in the calabash and starts pounding.		

TIME	ACTOR INITIALS	RECIPIENT INITIALS	WHAT HAPPENED	FOOD	Reactivity
			The two daughters came and help her in pounding it. She added water in the grains and kept after pounding		
2:40	BB		Baby sleeping		
2:45	BB		Sleeping		
2:50	FF		The father came in and sat on the local bed close to his father in-law		
2:55	FF	BB2	Father gives lolly pop to the boy	Lolly pop	
3:00	BB2		The young boy is drinking fura	Fura	
3:05	BB		Baby still sleeping		
3:10	MM		Mother lying down on a mat and baby sleeping		
3:15	MM		Mother pounding the sorghum into flour, sieve it and spread the flour		
3:25	BB		Sleeping		
3:30	BB		sleeping		
3:35	MM		Mother lying down on a mat		
3:40	BB		Sleeping		
3:45	BB		sleeping		
3:50	FF		Baby wakes up and the father took him		
3:55	FF		Father gives the baby to his mother and went to perform ablution he went out.		
4:00	MM	BB	Mother breast feeding the baby		
4:05	MM	BB	Mother put the baby on her feet with his legs open, he starts stooling after he finish. She		

TIME	ACTOR INITIALS	RECIPIENT INITIALS	WHAT HAPPENED	FOOD	Reactivity
			washes his body and pack the stool with shovel and throw it away		
4:10	BB		Baby playing around the house crawling		
4:15		BB	Baby holding his mother wrapper and she took him and back him		
4:20	MM	BB	Mother brings the baby down and give him water from the pot	water	
4:25	MM	BB2	Mother fetch pap from the bucket and give to the young boy in a cup to drink it himself		
4:30		BB	Baby crying and the mother breast feed him		
4:35	MM FW		The cows comes back and they tie them and give them water		
4:40	MM	BB	Mother fetch water in a wide rubber and put the baby inside to bath him		
4:45	MM	BB	Mother breast feeding the baby		
4:50	FW	DF	First wife give her daughter the beans she washed earlier for grinding. The first wife set the fire and put oil on a pan and starts frying the akara.		
4:55	MM	BB	Mother back the baby and starts washing the pot		
5:00	MM		Set the fire		
	MM	BB	Mother breast feed the baby		
5:05	BB2	BB	The young boy is eating beans cake made from beans paste. He cut small and give the baby	Beans cake	
5:10	FW		First wife finishes frying the beans cake give children 1, adult 2 and the remaining in a plastic bucket, her daughter took it out to sell		
5:15	MM	BB	Mother back the baby and continue with meal preparation,		

TIME	ACTOR INITIALS	RECIPIENT INITIALS	WHAT HAPPENED	FOOD	Reactivity
5:20	FF	MM	Father comes in with boiled yam and the mother share it among the children including the baby and kept the remaining in the room		
5:25	MM		Another pot is set on the fire		
5:30	MM		Mother brought out dried pepper, ginger and groundnut cake and pounds them together.		
5:35	FW	BB2	First wife give the young boy beans cake	Beans cake	
	MM	BB	Baby crying, first wife gave him the remaining pap (MNP pap left over)		
5:40	MM	BB	Mother breast feeding the baby		
5:45	FW		The wife is adding water into millet paste to make it watery and then add boiling water to make pap	pap	
5:50	MM		The soup preparation continues		
5:55	FW	BB	The first wife took the baby and back him		
6:00	MM		Mother takes the flour she made from sorghum and add water into it and stir it.		
6:05	MM		Takes dried baobab leaves and starts pounding it, until it is smooth, sieve it and kept aside		
6:10	BB		Baby sleeping		
6:15	BB		sleeping		
6:20	BB		Wakes up with crying and the first wife took him to his mother		
6:25	MM	BB	Mother gives baby water	water	
6:30	BB2	BB	The young boy is playing with the baby		

6:35	FF		Father comes in with pure water and gave them to the first wife		
6:40	MM		Mother open the pot and stir it, add sorghum flour to the require thickness		
6:45	FF	BB	Father with the baby sat under the tree		
6:50	MM	BB	Father brought the baby to the mother to breast feed him		
6:55	BB		Baby playing		
7:00	MM		Mother is re-stirring the food in the pot and close the pot		
7:05	MM	BB	Mother back the baby		
7:10	MM	BB2	Mother gave the little boy 1 beans cake	Beans cake	
7:15	MM	BB2	Mother put the pap for the little boy to drink	pap	
7:20	MM	BB	Baby crying and the mother gave him pap and water then back him		
7:25	BB		The baby is sleeping on his mother back		
7:30	MM	BB	Mother breast feeding the baby		
7:40	BB		The baby crying on his mother back		
7:45	MM		Mother takes the food to her in-laws, took the share of the first wife and her children to their room.<br>She kept hers and the husband share in her room		
7:50	BDF	BB	The first daughter of the first wife fetch in a bowl and eats, she give the baby little in his mouth	Tuwo sorghum and baobab leaves soup	
	FI MI		Mother is eating alone, and father in-law eating alone on his local bed		
7:55	MM	BB	Mother took the baby and wash his body, sat down and breast feed him and then back him		

	MM		She fetch food and starts eating	Tuwo sorghum and baobab leaves soup	
8:00	BB		Baby sleeping on his mother's back		
8:05			Still sleeping		
8:10			The young boy and the baby are still sleeping and the mother kept him on a local bed		

Primary Questions (Complete after you complete data collection):

From what you have seen observing THIS HOUSEHOLD, please answer the following questions in detail:

Question	Write your detailed response here
1. In this household, what would be the best way(s) to give nutritional supplements such as an MNP to young children to ensure compliance?<br><br>Why?	Through the mother because she takes care of the children. Although, the first and the second wife helps each other even the oldest daughter when she is at home
2. In this household, who is favored during meal preparation or feeding?<br><br>How is this favoritism expressed?	The husband because he has a larger portion of the food in a special bowl with a big fish.<br>The baby is also favoured because even before finishing the meal the fish was given to him and the mother is feeding him and being protected by the mother.
3. How could we promote the MNP supplement for this family that would attract their attention?<br><br>Who would they listen to?	Through their husbands because they all obey his instruction. He is in-charge of his household
4. In this household, what would be the best ways to	Through proper sensitization of women because as I can see they

promote the MNP but limit how much it would be shared?<br><br>Why?	are responsible in feeding the children not only feeding but total care of the children.
5. What other issues and concerns did you see with this family that are important for us to consider with regard to introduction of an MNP?<br><br>Why?	MNP administration is my major concern because as I can see, the caregivers/mothers do not have enough information about the product (sensitization)
6. Based on your experience observing this child today, do you believe reactivity negatively impacted your ability to see the food-related behaviors of the child?<br><br>Please explain your answer in detail.	Based on my experience observing this child today, reactivity does not impacted negatively on my ability to see food related behavior of the child to some extent. The only aspect is during the MNP administration, the first wife said to her, go and bring the pap that you add the medicine and give the baby, she looked at me and went and brought the pap. The child didn't want to take the pap, he will just sip and she will drop it and continue with breast feeding the child, is like she doesn't want to give the child.
